# A narrative review on the relationship between consumption of sugar-sweetened beverages and post-partum non-communicable diseases among women

**DOI:** 10.3389/fpubh.2025.1663238

**Published:** 2025-10-13

**Authors:** Zoha Imtiaz Malik, Syed Hassan Bin Usman Shah, Saira Zafar, Umar Farooq, Juweria Abid, Abdul Momin Rizwan Ahmad

**Affiliations:** ^1^Department of Human Nutrition and Dietetics, NUST School of Health Sciences, National University of Sciences and Technology (NUST), Sector H-12 Islamabad, Pakistan; ^2^The Kirby Institute, University of New South Wales, Sydney, NSW, Australia; ^3^Department of Public Health, Health Services Academy, Islamabad, Pakistan; ^4^Department of Nutrition and Dietetics, National University of Medical Sciences (NUMS), Rawalpindi, Pakistan; ^5^Department of Health Sciences, University of York, York, United Kingdom

**Keywords:** sugar-sweetened beverages, non-communicable diseases, post-partum, weight gain, weight retention, diabetes, mental health, cardiovascular disease

## Abstract

Recent years have seen an increased trend in the consumption of sugar-sweetened beverages (SSBs), which have been associated with weight gain and retention. These effects are also observed in women of reproductive ages, especially during the post-partum period, which enhances their risk of developing several non-communicable diseases later in life. The current narrative review aims to explore the relationship between SSBs consumption and post-partum weight gain and retention, by evaluating results garnered from observational and interventional studies, and reviews done previously on this topic. Literature search on Google Scholar and PubMed using the appropriate key words identified 8,653 articles, which after adjustment for timeline (2017–2025), and title, abstract and text screening for eligibility, resulted in 32 articles being selected. Evidence from the included articles suggests that SSBs result in weight gain due to their high caloric content, role in metabolic dysregulation and hormonal disruptions, exacerbating poor dietary intake and, increased blood glucose levels which ultimately lead to higher adiposity, both of which are risk factors for chronic diseases. Recommended solutions to reduce SSB intake in all population groups, particularly reproductive age women, include educational campaigns, SSB awareness using media, regulating SSBs sales through taxes and access policies, and making safe water available for the public. This review highlights the need to further delve into SSBs’ impacts on health and to focus on developing strategies to reduce their access and availability on a mass level. Barriers toward effective implementation of SSB regulation strategies also need to be addressed for more efficient and effective results.

## 1 Introduction

Post-partum non-communicable diseases (NCDs) are chronic conditions that emerge after and persist in the period after giving birth. In women the most prevalent post-partum NCDs include type 2 diabetes, obesity, cardiovascular diseases, and mental health disorders such as post-partum depression (PPD). These conditions may be influenced by dietary intakes, such as high consumption of sugar-sweetened beverages (SSBs), during the post-partum period (1). The prevalence of obesity is increasing rapidly all over the world, and women of reproductive age (15 to 49 years) are at greater risk as 47% women gain additional gestational weight than the recommended levels. This leads to subsequent complications such as development of chronic diseases and postpartum weight retention. It is hypothesized that eating behaviors and patterns may contribute to increased pregnancy weight gain and difficulty losing weight postpartum (2). Current evidence suggests that about 24% women of reproductive age are overweight in Pakistan, which makes the total prevalence of overweight and obese women around 38%. This indicates that a large number of women are at risk of excessive weight gain and retention during and after pregnancy, and poor dietary and lifestyle habits further amplify the risks (3). There is evidence suggesting the association between fried and high sugar foods, such as sugar-sweetened beverages (SSBs), with postpartum weight retention. Studies have found a weight gain of more than 5kgs during the postpartum period was found in women who had high intakes of sodas and sweet drinks. Additionally, consuming SSBs once a day, during the 3rd trimester and 30 days postpartum was associated with increased postpartum weight retention (4). In the US, about 27.3% of non-pregnant women of reproductive age are reported to consume one or more SSB, per day. Among pregnant women, 21.9% reportedly consume more than 1 SSB daily. The percentage of non-pregnant and pregnant women consuming 2 or more SSB per day, is 12.6 and 9.7%, respectively (5). In Sub-Saharan Africa, SSB consumption was found to be positively associated with high household income, with a study reporting 50.3% of 3,759 female participants to have consumed an SSB within the past 24 h prior to dietary data collection (6). The SSB consumption rates were low in South Asia, with mean intakes reported to be 0.7 servings per week, as per the 1990–2018 Global Dietary Database (GDD) analysis (7). The global trends have also found an increase in SSB consumption over the last 2 decades, more so in females than males. In 1990, the SSB consumption prevalence was 6.96% in young females aged 15–39 years, which has risen to 11.72% in 2021 (8).

Furthermore, there has been an increase in the SSB consumption frequency, with one in four non-pregnant women and one in five pregnant women consuming at least one sugar-sweetened beverage a day. These intake frequencies were also linked with lower education level, living in cities, being physically inactive, and smoking. Around 19.8% pregnant women in their 2^nd^ trimester consume more than 1 serving of SSBs which is further related to overall increase in caloric intake during pregnancy, increased gestational diabetes, preeclampsia and pre-term birth risk (5).

The greatest contribution to an individual’s total caloric intake comes from carbohydrates, therefore the type and amount of carbohydrates being consumed have a direct relation to weight gain. Foods high in processed carbohydrates and sugars lead to increased weight gain, while dietary fibers have a protective effect against obesity development. This is also true for postpartum weight as evidenced by studies suggesting that consuming SSBs at the 75^th^ percentile was associated with a weight gain of 2.08kgs during the postpartum period (9). Women with gestational diabetes, who had poor dietary habits including excessive consumption of ultra-processed foods, depicted a higher body mass index (BMI) during the postpartum period. This was particularly true for SSBs, which were associate with higher BMI scores at 6 and 12 months postpartum. An inverse relation between consumption of milk and milk products, and sugar-sweetened beverages was seen during the postpartum period, with the intake of dairy products dropping to 2–4% while SSBs consumption increased to 35.1% during this period. It is hypothesized that the dairy products may have been replaced by sugary fruit juices and sodas during this time (10). The main contributing factor to this finding is financial hardship, as healthier foods such as dairy products are expensive, processed foods such as SSBs are financially accessible for lower socioeconomic classes and therefore provide less expensive alternatives to healthy foods for these pregnant women. Additionally, new mothers may increase their intake of caffeinated beverages to fight off fatigue which further contributes to their overall SSB consumption. Some mothers may also be consuming fruit juices and sodas that are kept in the house for the children (11).

According to WHO’s dietary guidelines for pregnant women, no more than 10% of their caloric intake per day should come from SSBs. However, owing to the increased calorie demands and food cravings during pregnancy, pregnant females are more likely to consume more SSBs than non-pregnant females, which ultimately contributes to postpartum weight gain (12). This increased weight gain then results in a plethora of diseases related to elevated BMI levels such as diabetes, hypertension, insulin resistance and fatty liver during pregnancy and in the immediate postpartum period. Studies have found beneficial effects of replacing excessive carbohydrates with proteins, which also limits the intake of dietary sugars thereby reducing overall weight gain (13). It is evidenced that pregnant women consume 15.6 oz. of SSBs daily, contributing 176 calories to their total intake. Another factor to be considered when discussing SSB calories is the fact that these are liquid calories and individuals are more likely to consume higher levels of liquid calories as compared to those coming from solid foods, which showcases SSB tendency to cause excessive weight gain (14). Currently, 51% lactating women consume more sugars than the recommended intake which makes up 10% of their total caloric intake. Some studies suggested that this may be due to the perception that lactating women need to increase their fluid and energy consumption so some women may perceive SSBs are a source of free energy and a way to increase fluid intake (15).

As the issue of SSB consumption worsens with associated negative health consequences, several policy interventions can help reduce SSB consumption at the mass level. One such policy is the implementation of a national SSB tax, which may potentially reduce SSB purchases, particularly among lower socio-economic groups (16). Additionally, educational interventions, such as displaying nutrition and warning labels, or pictorial warnings on SSB packaging, pamphlet distribution and counseling in urban slums, can also lead to measurable decreases in SSB intake (17). Lastly, simultaneous effort to improve access to and availability of safe water, especially in resource poor communities, may support SSB substitution with water. In Pakistan, safe water usage is dependent upon education, income, awareness, and female empowerment (18).

While existing literature provides extensive insight on how SSBs are linked with weight gain and metabolic dysregulation, there is limited data on how SSBs consumption might impact post-partum weight retention. This further has critical consequences for post-partum recovery and chronic non-communicable disease risk. By generating comprehensive evidence specific to this critical time period, the current review aims to provide novel insights into how the consumption of SSBs during post-partum may impact long-term maternal health status, such as diabetes, mental health, and cardiovascular diseases.

## 2 Materials and methods

### 2.1 Study selection criteria

Inclusion criteria for this narrative review were original research articles that reported on sugar-sweetened beverages intake and maternal health outcomes. These included observational and interventional studies, including cross-sectional, quasi-experimental, randomized-control trials, and longitudinal cohort study designs. The studies included were published from 2017 to 2025, focusing on post-partum NCDs. For the purpose of this review, we defined post-partum NCDs as chronic health conditions arising during and then persisting after the post-partum period, such as type 2 diabetes, obesity, CVDs and PPD. Exclusion criteria included any studies conducted on men and children, as well as, reviews, abstracts, and commentaries.

### 2.2 Search strategy

A comprehensive search was conducted on two databases; PubMed and Google Scholar, by employing the keywords: “sugar-sweetened beverages,” “postpartum,” “maternal health,” “weight retention,” and “non-communicable diseases.” The initial search yielded 8,653 records, and removal of any duplicates, title and abstract screening, and removal of non-relevant studies was conducted. Furthermore, full-text analysis of eligible records was done, which then resulted in 32 articles that met the inclusion criteria and formed the basis of this narrative review.

### 2.3 Data extraction and preparation

Two independent reviewers retrieved full texts of all eligible articles and examined them against the inclusion criteria. A standardized data extraction form was used to collect article information on author(s), year of publication, title, country, study design, participant characteristics, study results, and maternal health outcomes.

### 2.4 Quality assessment of included studies

The methodological quality of the study was assessed for its study design, sample size, and appropriateness of maternal health outcomes. Any uncertainties regarding study eligibility or quality were resolved with discussion among review authors. The Scale for the Assessment of Narrative Review Articles (SANRA), a validated tool for quality assessment of studies to be included in narrative reviews, was used for this purpose. It was developed by Baethge et al., to assess the quality of narrative reviews (19). It is a six item scale including, justification of the article’s importance, statement of concrete aims, description of the literature search, referencing, scientific reasoning, and appropriate presentation of data, each of which is scored as 0 = low, 1 = moderate, 2 = high quality. Two reviewers independently reviewed each study against the mentioned domains and gave a score. The mean SANRA score for each study was calculated and scores of 0–4 were considered poor quality, 5–8 moderate, and 9–12 good.

## 3 SSBs consumption, postpartum weight gain and risk of diabetes

SSBs consumption is increasing across all age groups, with 18.4% Pakistani university students aged 18 to 35 years reporting consuming at least one SSB per day. The most commonly consumed SSBs were coke (41.6%), and packaged juices (31.2%) (20). The International Diabetes Federation estimates that 31.4% of adults in Pakistan have diabetes, which equals to approximately 34.5 million individuals (21). Recent evidence has suggested a strong positive association between sugar-sweetened beverages and diabetes mellitus, along with the development of several other cardio metabolic risk factors that have negative health consequences. Several longitudinal cohort studies have found a 16 to 18% increased type 2 diabetes risk in adults consuming SSBs, while replacing one serving of SSB with a non-sweetened beverage such as water reduced the risk by 2–10% (22). Despite this data being compelling due to large sample size and prospective follow-up findings, they may not relate to the South Asian populations where dietary and beverage consumption patterns differ, and beverages like sweetened teas are part of culture which may be difficult to completely cut out. Women of reproductive age have additional risk of developing type 2 diabetes in life, if they had gestational diabetes or gained more than the recommended weight during pregnancy and postpartum. It was evidenced that a postpartum weight gain of 4.5 kg increased the risk of type 2 diabetes two times. Additionally, women with gestational diabetes, were at an even higher risk, as every 5 kg weight gain was related to a 27% increase in type 2 diabetes risk (23). This highlights the vital link between post-partum weight gain and diabetes, but the study focuses mainly on gestational diabetes mellitus may limit generalizability of results to all women. As the global consumption of SSBs increases, they have also gained attention in relation to weight gain and diabetes risk in reproductive age women. Research has shown 21.9% women consume at least 1 SSB per day and pregnant women consuming SSBs had an overall poor diet quality, associated with higher risk of postpartum weight gain and diabetes mellitus. The reason SSBs contribute to both of these is due to their high glycemic load which raises blood glucose levels, decreased satiety leading to increased calorie consumption from other foods, and its association with pancreatic beta cell dysfunction, and insulin resistance (24). This study does provide strong evidence supporting the role of SSBs in metabolic dysregulation, however, as dietary data was respondent-dependent, recall bias and cultural dietary variations serve as potential limitations (Table 1).

**Table 1:** SSB consumption, postpartum weight gain and risk of diabetes.

Study reference	Country	Study participants	Study design	Study results	Odds ratio (95% CI)	Conclusion	Interpretation
Drouin-Chartier et al. (22)	USA	34,224 men & 158,128 women across 3 large cohorts	Prospective cohort (dietary intake assessed every 4 years)	>0.5 serving/day SSB increased diabetes risk by 16%	Not reported	Higher SSB intake linked to type 2 diabetes	Large, high-quality cohorts; strong evidence for diabetes risk; generalizable to high-income populations but not postpartum-specific
Davis et al. (23)	USA	1,035 women with history of GDM	Prospective cohort (2 years follow-up)	≥2 servings per week SSB increased odds of postpartum weight retention	1.95 (1.22–3.11)	Reducing SSB intake may reduce weight retention	Directly relevant to postpartum women; suggests even moderate SSB intake contributes to weight retention
Yong et al. (24)	Malaysia	452 pregnant women	Prospective cohort	SSBs (especially fruit juice) increased odds of GDM	0.98 (0.97–0.99)	SSBs are associated with GDM	Adds LMIC perspective; highlights fruit juices (often perceived as “healthy”) as risk factor
Kay et al. (25)	USA	217 low-income Black mothers	Longitudinal observational	38% consumed SSBs ≤1.5 servings/day; overall poor diet quality	Not reported	Poor postpartum diet quality, obesogenic patterns	Emphasizes socioeconomic disparities; SSBs part of broader unhealthy diet
Jackson et al. (26)	USA	5.3 M birthing individuals	Quasi-experimental (tax policy evaluation)	SSB taxes decrease GDM risk by 41.4% and lower gestational weight gain by 7.9%	Not reported	Policy interventions improve maternal outcomes	Strong population-level evidence; unique intervention design; suggests taxation can directly improve perinatal health
Dalrymple et al. (27)	UK	667 obese women	RCT (diet & physical activity intervention during pregnancy)	48% retained more than their pre-pregnancy weight	Not reported	Targeting modifiable determinants prevents weight retention	Not SSB-specific but reinforces role of diet & lifestyle in postpartum outcomes
Most J et al. (43)	USA	37 obese pregnant women	Prospective cohort (12 mo postpartum)	57% gained weight; linked to incresed calorie intake	Not reported	Energy intake determines postpartum weight retention	Small sample; not SSB-specific but relevant for energy balance context

The postpartum period significantly impacts a woman’s health, and around 58.5 to 80% women may be obese during the postpartum period. Reasons may include the discontinuation of healthier dietary patterns that were followed during pregnancy, economic and time constraints, lack of access and availability of healthy foods, and presence of postpartum mental health issues such as depression. One variable of interest is the empty calories, which 85% of these women consume in the form of SSBs. They provide at least 275cals per day which is more than the recommended 258cals that should come from all extra sugars (25). This emphasizes how replacement of healthier food options with SSBs, particularly in low and middle income countries. However, the study was highly specific and limited to black, non-Hispanic mother, which may limit applicability across cultures. SSBs also constitute the largest source of added sugars for pregnant females, and make more than half of the overall added sugars consumed daily. These increased SSB intakes are then associated with negative maternal as well as neonatal complications. Gestational and type 2 diabetes as well as excessive weight gain have all been associated with increased SSB intakes (26). Excessive weight gain itself is an independent risk factor for type 2 diabetes development, and while the average time it takes for a woman to return to her pre-pregnancy weight is 6 months, those with excessive gestational weight gain tend to retain more than 5kgs during the postpartum period. This is linked with a higher BMI which then contributes to several cardio metabolic issues including diabetes mellitus (27). However, to establish causal mechanistic pathways between intervention trials are needed, which are still limited among post-partum females.

## 4 Postpartum weight gain and mental health

While the main aim of our review focuses on the relationship between SSBs, weight retention, and NCDs, this section explores how post-partum weight retention may contribute to mental health issues, such as depression, which in turn impact dietary behaviors, including SSB consumption (Table 2). The influence of gestational and postpartum weight gain on maternal mental health is not well established. Body mass index (BMI) and prenatal depression have been positively associated, with a greater risk of depression in those who had a high BMI. Additionally, those with depression were more likely to gain weight during pregnancy and postpartum (28). While this highlights a vulnerable population group, the adolescent sample may limit generalizability to older women. The relation between weight gain and depression can be explained by the inflammatory hormone secreted by adipose tissues which lead to chronic inflammation associated with depression. Furthermore, body image issues due to pregnancy weight gain can also produce feelings of depression and anxiety (29). Another mechanism that explains the association between depression and obesity is the dysregulation of hypothalamic–pituitary–adrenal (HPA) axis, which occurs in both these conditions. This disturbance of the HPA axis leads to increased cortisol levels in both obese and depressed individuals. The HPA axis is altered significantly during pregnancy and takes months after a woman has given birth to get back to normal functioning. This is crucial in explaining postpartum depression and weight retention during this period (30). The HPA axis provides a significant link between obesity and depression, highlighting how hormonal dysregulation may influence both postpartum depression and weight retention. However, most of the evidence is generated from studies that did not include a postpartum specific cohort. Additionally, longitudinal studies focusing on cortisol pathways during pregnancy and postpartum are limited, making it difficult to develop causality. Moreover, psychological factors such as sleep disturbances, stress, and lack of social support during postpartum, may act as confounders in the HPA axis activity.

**Table 2:** Postpartum weight gain and mental health.

Study reference	Country	Participants	Methodology	Results	Odds ratio (95% CI)	Conclusion	Interpretation
Cunningham et al. (28)	USA	505 pregnant adolescents (14–21 yrs)	RCT with physical and educational assessments during pregnancy up till 6 months postpartum	50% had excessive gestational weight gain (GWG), linked to higher postpartum depression	2.5 (1.1–5.8)	Excessive GWG in adolescents increased risk of postpartum depression	Highlights unique risk in adolescents; adds age-specific vulnerability but limited by short 6-month follow-up
Dayan et al. (29)	Iran	223 healthy women (BMI 18.5–30)	Prospective cohort; GWG measured at 2^nd^ and 3rd trimester; post-partum depression (PPD) assessed at 6–8 weeks postpartum	32.2% had PPD; 3rd trimester GWG was associated with PPD	1.17 (1.04–1.32)	Management should consider impact of late-trimester weight gain	Supports trimester-specific vulnerability; cultural context relevant but convenience sampling limits generalizability
Ertel et al. (30)	USA	2,112 women (1,686 followed postpartum)	Prospective cohort; self-reported pre-pregnancy weight & GWG; depression scale assessment was done at mid-pregnancy and 6 months postpartum	10% prenatal and 9% postpartum depression; pre-pregnancy BMI > 30 increased PPD risk	1.27 (1.02–1.58)	Pre-pregnancy obesity linked to higher depressive symptoms	Strong sample size; shows pre-pregnancy weight effect beyond GWG; reliance on self-reported weight is a limitation
Yu et al. (31)	USA	424 postpartum women (after exclusions)	3-arm RCT (gestational vs. gestational and postpartum vs. placebo interventions)	Postpartum depressive symptoms and self-weighing linked with unhealthy eating	Not reported	Mental health interventions may improve eating & weight outcomes	Rare intervention trial; integrates mental health & behavior change; limited by attrition and generalizability
Baruth et al. (33)	USA	5,000 women	Survey across 12 months postpartum (dietary inflammatory index (DII), PPD, weight retention)	DII score not a predictor of postpartum weight retention	Not reported	Dietary quality not significant for PPD-related weight outcomes	Suggests diet alone insufficient; large sample strengthens results but survey nature reduces causality
Wu et al. (35)	Taiwan	318 women (>20 yrs., singleton pregnancies)	Cross-sectional; BMI, GWG, depression, and emotional eating assessed postpartum	23.9% had PPD; excessive GWG & emotional eating linked with PPD	Not reported	Negative emotions and overeating perpetuate weight retention & PPD	Strong link between emotional eating and depression; but cross-sectional design prevents causal inference

Postpartum is an emotionally vulnerable time for women which makes them more prone to experiencing stress and depressive episodes. This mental and emotional dysfunction can be associated with emotional and disordered eating patterns, which may lead to unhealthy food consumption and other poor lifestyle habits. Both of these have been associated with decreased fruits and vegetables intake, as well as elevated body weight levels during 12 to 18 months after postpartum (31). However, eating behaviors and depression have a bidirectional relationship, therefore it is difficult to assess if the disordered eating patterns stemmed from depression or if the poor eating habits may have exacerbated psychological symptoms. Hence, interventions should focus on both correcting eating patterns while addressing underlying psychological issues, particularly in the postpartum period. Studies have found that pre-pregnancy BMI, rather than gestational weight gain, is more strongly associated with antenatal depression and other mental health disorders. There appears to be little to no association between weight gained during pregnancy and depressive symptoms. This can be explained by the fact that women believe pregnancy is a crucial period in their lives and do not think much of the weight and body changes they may experience during these months. However, as mentioned earlier, if they fail to lose the desired amount of weight postpartum, it may lead to the development of depressive symptoms in these women (32). This highlights the role of psychological and cultural perceptions of pregnancy, which may put undue pressure on females postpartum to immediately lose weight. This may add to their depressive symptoms, which is then associated with weight retention, making it difficult to attain pre-pregnancy weight. In Asian and South Asian cultures, the social pressures and limited postpartum support may add to body image issues, which may amplify the mental health impact of weight retention.

Other weight related variables that were found to be associated with increased PPD include, higher postpartum BMI, minimal weight loss at the 6 months postpartum mark, and retaining significant weight after giving birth (33). Conversely, women who are suffering from mental health issues are more likely to engage in unhealthy lifestyle activities that may prevent postpartum weight loss. It has been evidenced that PPD in women can increase the risk of postpartum weight retention by at least 5kgs. Furthermore, stress induced eating disorders such as binge eating, can lead to further difficulty in losing weight (34). This bidirectional relationship adds to the complexity of addressing mental health issues during this period. In addition to directly contributing to unhealthy behaviors such as sedentary lifestyles and poor eating habits, depression can also foster harmful coping strategies such as binge eating, which may further contribute to weight retention. Therefore, contributing to the vicious cycle of depression and weight gain. There is evidence suggesting higher intakes of sugar and fats among women with diagnosed postpartum depression. On the contrary, women who were obese prior to getting pregnant may also experience restrictive eating in an effort to quickly lose the gained gestational weight, which is then related to increased depression incidence in these women (35). These findings suggest the multifaceted relationship between diet, weight retention, and depression. While poor eating such as high sugar and fat intake may worsen depressive symptoms through inflammatory and neurochemical pathways, restrictive dieting may also have negative psychological impacts on women. This duality highlights the need to tailor nutrition interventions to promote both weight management and psychological recovery postpartum.

## 5 Postpartum weight gain and cardiovascular disease

Cardiovascular disease (CVDs) is one of the leading mortality causes in women, and those of reproductive age are at excessive risk for CVD development (Table 3). It is mostly associated with additional weight gain, and Pakistani females are already at a higher risk of being obese due to its high prevalence in the country (36). Around 25% of women who give birth are unable to lose weight for the first 1 to 2 years after delivery, which is associated with subsequent obesity and CVD risk factor presentation. Excessive postpartum weight retention is positively related with abdominal adiposity in women, which is a significant contributor to CVDs as evidenced by the fact that for each 1 point increase in a woman’s BMI from pre-pregnancy to 18 months postpartum, is linked to higher CVD risk (37). Further studies have reported a postpartum weight retention of more than 3kgs between consecutive pregnancies, increases the risk of gestational diabetes, and hypertension, both of which are important cardio-metabolic factors in CVD occurrence (1). The implication that even a modest weight retention of 3kgs may elevate metabolic disease risk, highlights how postpartum weight retention may set the foundation for future cardio-metabolic complications across pregnancies and later in life as well.

**Table 3:** Postpartum weight gain and cardiovascular disease.

Study reference	Country	Participants	Methodology	Results	Odds ratio	Conclusion	Interpretation
Kirkegaard et al. (37)	Denmark	47,966 women (18 months postpartum follow-up)	Large prospective cohort; weight and health outcomes assessed at 6 & 18 months postpartum	1,321 CVD cases; risk increased up to 38% in women who gained within recommendations but failed to return to pre-pregnancy weight; 70% increased risk in underweight women with insufficient gain	Not reported	Both weight retention and inadequate weight gain linked to long-term CVD risk	Very strong sample size; highlights double risk (excessive and insufficient gain); limited by reliance on registry data without lifestyle details
Soria-Contreras et al. (38)	Mexico	361 women followed up to 6 years postpartum	Secondary analysis of a pregnancy cohort; weight tracked at multiple postpartum points	6% retained weight, 13.9% gained, 19.9% had both; all groups had high BMI, waist circumference, and insulin resistance	Not reported	Postpartum weight retention/gain resulted in higher long-term obesity & metabolic risk	Adds long-term perspective; smaller sample size; attrition may bias findings
Wahabi et al. (39)	Saudi Arabia	115 women, 12 months postpartum	Longitudinal cohort; weight retention categorized into <3 kg, 3–7 kg, >7 kg	35% retained >7 kg, linked with a 13% higher risk of metabolic syndrome	Not reported	Higher weight retention was associated with increased prevalence of cardio-metabolic risk factors	Small sample but culturally significant; demonstrates dose–response trend between retention and risk

Another mechanism explaining the positive association between postpartum weight gain and cardiovascular diseases, is the transference of peripheral fat to the abdominal adipose tissues after pregnancy, which is directly linked to an increased CVD risk as most of this fat is accumulated around the viscera. Hence, both postpartum weight gain and retention are responsible for worsening metabolic profiles in women (38). This can help explain why women are at 5 time greater risk of developing both fatal and not fatal CVDs during 40 to 60 years, as compared to a 2 times increased risk in men. Furthermore, the triglyceride and blood lipid levels increase during pregnancy, particularly in the 3^rd^ trimester, and then fall during postpartum. However, after 1 year postpartum the HDL levels dropped even below than the pre-pregnancy levels, which is an independent risk factor for CVD development. On the contrary, postpartum weight loss and maintenance helps improve the cardio metabolic profiles and reduce the risk of CVDs (39). This underscores the importance of tailoring interventions not just for weight management but also to improve lipid profiles postpartum. These implications are of great importance as different population group exhibit different risks of cardiovascular diseases, and postpartum lipid profile alterations may have greater negative consequences.

Pregnant women belonging to underprivileged populations are at a greater risk of CVD related morbidity and mortality, due to the high prevalence of CVD risk factors in these communities, as well as inaccessibility to adequate management of these conditions (40). This disproportionate burden among the lesser privileged females highlights the role of social determinants of health, as lack of postpartum healthcare, nutrition awareness, and limited financial and physical access to health services all contribute to the CVD burden in women. In such communities, weight retention is not just a matter of personal choices, but a structural challenge emerging from intergenerational poverty and poor health. The American Heart Association suggests close monitoring of CVD risk factors during the 1^st^ year of postpartum, as gestational weight gain, diabetes and hypertension developed during pregnancy has significant impact on the cardio-metabolic profile of these women. Therefore, the AHA recommends consistent and comprehensive evaluation of women until 1 year of postpartum to assess the occurrence of CVD risk factors during this period. Women with CVDs make a high risk group for adverse pregnancy outcomes and require longitudinal care and management during the postpartum period. Some impactful strategies include provision of initial postpartum care, development of transitional clinics, awareness raising, and lifestyle modification interventions (41). Despite these recommendations, 40% of postpartum women did not attend any clinics to assess their cardio-metabolic risk factors, and only 57% of those who give birth were given appropriate lifestyle related education. The rate of postpartum counseling provision varied immensely in terms of social status, putting underprivileged women at a greater risk of not receiving the necessary care needed to prevent and manage CVD risk factors including postpartum weight gain. The postpartum period can help a woman achieve and maintain good cardio-metabolic health, if given the right opportunities and awareness (42). These findings demonstrate a major gap in postpartum care, especially in lower and middle income countries, where post-delivery follow-up and counseling services are not widely available. Strengthening postpartum education and support can play a positive role in reducing weight retention and associated CVD risk.

Our review has certain limitations, including the heterogeneity of included studies on SSB consumption, and associated postpartum weight gain and metabolic diseases. The included studies differ in study design as some are cross-sectional while others are interventional. The populations studied also vary according to age, ethnicity and socio-economic and demographic backgrounds, which may impact findings. Additionally, outcome measures for these studies ranged from weight retention to BMI to various other metabolic markers. Such variation may limit direct comparability and weaken causal inferences. Furthermore, some studies relied on self-reported data from participants which maybe prone to recall and reporting bias. However, despite these limitations, the overall evidence reviewed suggests positive associations between SSB consumption during post-partum period and adverse long-term health consequences, highlighting the importance of reducing their consumption during this critical window.

## 6 Conclusion

The current review concluded that the consumption of sugar-sweetened beverages post-partum poses significant risks in terms of development of several non-communicable diseases. It is strongly associated with obesity and subsequent diabetes risk as evidenced by longitudinal and cohort studies. It is further associated with cardiovascular diseases through pathways involving weight retention, alteration of blood lipid profiles, and increased abdominal obesity. In contrast, the association of SSBs with deterioration in mental health remains less consistent, despite biological plausibility, and requires further robust research. Overall evidence suggests targeted interventions should be tailored toward reducing SSB intake in women, raising awareness regarding their ill-effects and prioritizing research into emerging pathways. Policy measures such as SSB taxation, restricting their availability in super-markets, schools, and workplaces, and public health awareness campaigns can all promote healthier beverage choices, and mitigate SSB associated metabolic and non-communicable diseases.
